# Proteolytic activity of *Triatoma infestans* saliva associated with PAR-2 activation and vasodilation

**DOI:** 10.1590/1678-9199-JVATITD-2020-0098

**Published:** 2021-03-08

**Authors:** Karla A. Oliveira, Ricardo J. S. Torquato, Daniela C. G. Garcia Lustosa, Tales Ribeiro, Bruno W. L. Nascimento, Lilian C. G. de Oliveira, Maria A. Juliano, Thaysa Paschoalin, Virginia S. Lemos, Ricardo N. Araujo, Marcos H. Pereira, Aparecida S. Tanaka

**Affiliations:** 1 Department of Biochemistry and Pharmacology, Federal University of Piauí, Teresina, PI, Brazil.; 2 Department of Biochemistry, Federal University of São Paulo (Unifesp), São Paulo, SP, Brazil.; 3 Department of Pharmacology, Institute of Biomedical Sciences, Federal University of Minas Gerais (UFMG), Belo Horizonte, MG, Brazil.; 4 Department of Parasitology, Institute of Biomedical Sciences, Federal University of Minas Gerais (UFMG), Belo Horizonte, MG, Brazil.; 5 Department of Biophysics, National Institute of Pharmacology and Molecular Biology (INFAR), Federal University of São Paulo (Unifesp), São Paulo, SP, Brazil.; 6 Department of Physiology and Biophysics, Institute of Biomedical Sciences, Federal University of Minas Gerais (UFMG), Belo Horizonte, MG, Brazil.; 7 National Institute of Science and Technology in Molecular Entomology (INCT-EM), Rio de Janeiro, RJ, Brazil.

**Keywords:** Triapsin, Vasodilation, PAR-2 receptor, Nitric oxide, Endothelial cells

## Abstract

**Background:**

*Triatoma infestans* (Hemiptera: Reduviidae) is a hematophagous insect and the main vector of *Trypanosoma cruzi* (Kinetoplastida: Trypanosomatidae). In the present study, the authors investigated whether a serine protease activity from the saliva of *T. infestans* has a role in vasomotor modulation, and in the insect-blood feeding by cleaving and activating protease-activated receptors (PARs).

**Methods:**

*T. infestans* saliva was chromatographed as previously reported for purification of triapsin, a serine protease. The cleavage activity of triapsin on PAR peptides was investigated based on FRET technology. Mass spectrometry was used to analyze the sites of PAR-2 peptide cleaved by triapsin. NO measurements were performed using the DAN assay (2,3-diaminonapthalene). The vasorelaxant activity of triapsin was measured in vessels with or without functional endothelium pre-contracted with phenylephrine (3 µM). Intravital microscopy was used to assess the effect of triapsin on mouse skin microcirculation.

**Results:**

Triapsin was able to induce hydrolysis of PAR peptides and showed a higher preference for cleavage of the PAR-2 peptide. Analysis by mass spectrometry confirmed a single cleavage site, which corresponds to the activation site of the PAR-2 receptor. Triapsin induced dose-dependent NO release in cultured human umbilical vein endothelial cells (HUVECs), reaching a maximum effect at 17.58 nM. Triapsin purified by gel-filtration chromatography (10^-16^ to 10^-9^ M) was applied cumulatively to mouse mesenteric artery rings and showed a potent endothelium-dependent vasodilator effect (EC_30_ = 10^-12^ M). Nitric oxide seems to be partially responsible for this vasodilator effect because L-NAME (L-NG-nitroarginine methyl ester 300 µM), a nitric oxide synthetase inhibitor, did not abrogate the vasodilation activated by triapsin. Anti-PAR-2 antibody completely inhibited vasodilation observed in the presence of triapsin activity. Triapsin activity also induced an increase in the mouse ear venular diameter.

**Conclusion:**

Data from this study suggest a plausible association between triapsin activity mediated PAR-2 activation and vasodilation caused by *T. infestans* saliva.

## Background

*Triatoma infestans* (Hemiptera: Reduviidae) is a hematophagous insect and the main vector of *Trypanosoma cruzi* (Kinetoplastida: Trypanosomatidae), the agent of Chagas’ disease in Latin America [1]. The saliva from hematophagous arthropods has a variety of molecules to overcome host hemostatic responses, such as vasoconstriction, blood coagulation and platelet aggregation, and help the acquisition of the blood meal [2]. Accordingly, an analysis of the *T. infestans* sialome [3] revealed a high content of lipocalins (a heterogeneous group of proteins, mainly carriers of small ligands in vertebrates and invertebrates) and a number of other putative secreted proteins with potential anti-hemostatic activity. The identification and characterization of antiplatelet [4, 5] and anticoagulant [6] activities from the saliva of *T. infestans* have been described. In addition, vasodilation activity was shown from extracts of *T. infestans* salivary glands by an unknown mechanism [7]. Triapsin is a serine protease produced and stored in the salivary gland of *T. infestans*, which is released in an active form in the saliva [8]. The protease was previously characterized and showed a high specificity for Arg at P1 position [8], but no target or function was identified. 

In this work, we investigated if a preparation of *T. infestans* saliva containing triapsin has a role in vasomotor modulation by cleaving and activating protease-activated receptors (PARs). PARs are a subfamily of G protein-coupled receptors (GPCRs) with seven transmembrane domains and are activated by serine proteases [9-12]. A specific endogenous activator for PAR-1, PAR-3 and PAR-4 is thrombin, while PAR-2 is activated by trypsin, tryptase and coagulation factors VIIa and Xa but not thrombin [11-13]. PARs, especially PAR-1 and PAR-2, are expressed in various tissues/cells in the mammalian body and are involved in a number of biological events [14-22]. PAR-2 has been shown to trigger vasodilation in most studied arteries [23-26] and previous works indicated that PAR-2 is also located on the smooth muscle cells of mouse renal arteries and mediate contraction, suggesting that PAR-2 may mediate appropriate vascular responses to diverse stimuli [27]. The present study provides experimental data to suggest an association between triapsin protease activity, PAR-2 activation and vasodilation caused by *T. infestans* saliva observed during insect feeding on blood.

## Methods

### *Triatoma infestans* and saliva collection

Triatomine colonies were reared at a temperature of 28±1°C, relative humidity of 60-70% and 12 h/12 h light/dark cycle at the Laboratory of Physiology of Hematophagous Insects in Universidade Federal de Minas Gerais (UFMG), Belo Horizonte, Brazil. For colony maintenance, the insects were regularly fed on Wistar rats or Swiss mice. The saliva was collected from adults (females and males) using a pipette tip as previously described [8] and stored at -80°C until use.

### Animals

For intravital microscopy experiments, approximately twelve-week-old hairless mice (HRS/J) were obtained from the Animal Care Facilities, Department of Parasitology, ICB/UFMG. For *ex vivo* vasodilation assays, eight- to twelve-week-old Swiss mice (male) were obtained and housed under standard conditions with free access to commercial food and water. Animal experiments were approved by the UFMG Ethical Committee on Animal Use, under protocol number 115/2011.

### Cell culture

The human umbilical vein endothelial cell (HUVEC) line was provided by Julio Scharfstein from Federal University of Rio de Janeiro. Cells were grown to approximately 90% confluence in RPMI 1640 medium, pH 7.2, supplemented with 10 mM N-2-hydroxyethylpiperazine-N’-2 ethanesulphonic acid (HEPES), 24 mM sodium bicarbonate, 10% heat-inactivated fetal calf serum (FCS; Gibco, Minneapolis, MN, USA) and 40 μg/mL gentamicin sulfate (Hipolabor Farmacêutica, Sabará, MG, Brazil) and maintained at 37(C in a humidified incubator with 5% CO_2_ atmosphere. Cells from passage 2-8 were used in all experiments.

### Triapsin purification

Purification of native triapsin from *T. infestans* saliva was performed according to Amino et al. [8]. Approximately 50 µL of saliva, diluted in 550 µL of Milli Q water, was chromatographed in a HiTrap Q FF column (GE Healthcare Life Science) equilibrated in 20 mM Tris-HCl, pH 8.0. The activity was eluted with a gradient up to 1.0 M NaCl in five volumes of the column. Fractions from the peak containing the highest activity were pooled and mixed with the same volume of 3.4 M (NH_4_)_2_SO_4_ and applied into a Source 15 PHE (Phenyl) column (GE Healthcare Life Science) equilibrated with 20 mM Tris-HCl, pH 8.0, 1.7 M (NH_4_)_2_SO_4_. The activity was eluted with a gradient up to 20 mM Tris-HCl, pH 8.0. Fractions containing activity were pooled and concentrated using a 10,000 NMWL Centrifugal Filter Device (Amicon). For *in vivo* and *ex vivo* experiments, the enzyme was purified in a Superdex 75 column (GE Healthcare Life Science) using PBS buffer. Fractions containing activity were pooled, concentrated, quantified for proteolytic activity, and stored at -20°C until use. A sample of 3% of the concentrated pool formed with the eluted activity from Source 15 PHE column was loaded on a 12% SDS-PAGE gel and stained in 0.1% silver nitrate solution. About 148 μU of triapsin were applied to Source 15 PHE and 46.5 μU were applied to Superdex 75 and 7.1 μU recovered after this step of purification.

### Enzyme activity measurements and protein quantification

Triapsin activity in saliva or purified fraction was estimated by the release of p-nitroaniline from the synthetic substrate H-D-Ile-Pro-Arg-pNA (S2288) supplied by Chromogenix [8]. The assays were performed in 96-well plates in 50 µL of 100 mM Tris-HCl buffer, pH 8.0, and 0.4 mM of chromogenic substrate (S-2288) and measured at 405 nm in a Microplate Spectrophotometer (Gene5_BioTek® Instruments). One enzymatic unit was defined by the release of 1 mmol of p-nitroaniline per min at 37°C at pH 8.0. The concentration of purified triapsin was estimated using a Nanodrop 2000 (Thermo) assuming one unit at A280 nm = 1 mg/mL. The concentration of total protein in saliva was determined by the Bradford dye-binding assay using bovine serum albumin as a standard [28]. For inhibitory assays, serine protease inhibitor (SBTI- Soybean trypsin inhibitor from Sigma-Aldrich) was tested with the saliva in the presence of the substrate (S2288), as described above. Inhibitor concentrations were used in all experiments to produce higher than 50% inhibition of triapsin activity. All measurements were performed considering limited sample availability. 

### Cleavage of PAR peptide

The cleavage activity of triapsin on PAR peptides was investigated based on Fluorescence Resonance Energy Transfer (FRET) technology. Peptide sequences that span the cleavage sites for human PAR-1, -2, -3, and -4 activation were according to Coughlin [29] as follows: PAR-1_TLDPR↓SFLLRN, PAR-2_SSKGR↓SLIGKVDGT, PAR-3_TLPIK↓TFRGAPPNS, PAR-4_LPAPR↓GYPGQVCAN. The peptides were synthesized with the fluorescent group Abz (o-aminobenzoic acid) and the quenching group EDDnp (ethylenediamine-2,4-dinitrophenyl) at the N- and C-terminus ends, respectively (GenOne Biotechnology). Assays were performed with purified triapsin and 125 μM each peptide in 100 mM Tris-HCl buffer pH 8.0 at 37°C in a Microplate Spectrophotometer (Gene5_BioTek® Instruments) and read at 320 nm and 420 nm for excitation and emission, respectively. 

### Analysis of PAR-2 peptide cleavage by mass spectrometry

Mass spectrometry was used to analyze the sites of PAR-2 peptide cleaved by triapsin. For that, PAR-2 peptide was incubated with *T. infestans* purified triapsin overnight at 37(C in 100 mM Tris-HCl buffer pH 8.0. The scissile bond of hydrolyzed peptides was identified by the isolation of fragments using analytical HPLC followed by determination of their molecular masses with an LCMS-2020 instrument equipped with an electrospray ionization (ESI) probe (Shimadzu, Tokyo, Japan).

### Cell viability assay

Cell viability was tested by using MTT assay (M6494-ThermoFisher Scientific), according to the manufacturer’s protocol. For this assay, human umbilical vein endothelial cells **(**HUVECs) were grown in 96-well plates in triplicate (5 x 10^3^ cells/well) and then incubated with HBSS (Hanks' Balanced Salt Solution) or triapsin (17.85 nM) for 50 to 60 min, at 37˚C. The result is the mean ± SEM of three independent experiments. 

### Nitric oxide (NO) measurements

Nitric oxide (NO) measurements were performed according to Misko et al. [30] and Kleinhenz et al. [31] using the DAN assay (2,3-Diaminonapthalene), based on the reaction of 2,3-Diaminonapthalene with nitrite under acidic conditions to form 1-(H)-naphthotriazole, a fluorescent product. For this assay, HUVECs grown in 96-well plates in triplicate (5x10^3^ cells/well) were washed two times with warm HBSS buffer and stimulated with different concentrations of triapsin (0 to 35.7 nM), or 50 nM trypsin (positive control) in 60 µL of HBSS for 50 to 60 min at 37˚C. Acetylcholine (10^-5^ M) was used to determine the maximum effect of NO production. After, 50 µL of the supernatant was transferred to a new 96-well plate and incubated with nitrate reductase (14 mU) and NADPH (40 µM) at 25°C for 30 min to reduce nitrate (NO_3_) to nitrite (NO_2_). Then, DAN at a concentration of 0.05 mg/mL was dissolved in 0.62 N HCl, and 10 µL was added to each well for 10 min at room temperature. After, 5 µL of 2.8 N NaOH was added to each well, and the plate was read on a Microplate Spectrophotometer (Gene5_BioTek® Instruments, Inc.) at 365 nm excitation and 450 nm emission. Standard curves were made with sodium nitrite ranging from (0.3-10 µM) in HBSS.

### Small mesenteric artery preparation and mounting

Male Swiss mice were killed by decapitation. The viscera were exposed, and a proximal segment of the small bowel was removed and pinned in a dissecting dish containing physiological salt solution (PSS) of the following composition: 119 mM NaCl, 4.7 mM KCl, 0.4 mM KH_2_PO_4_, 14.9 mM NaHCO_3_, 1.17 mM MgSO_4_, 2.5 mM CaCl_2_ and 5.5 mM glucose. Branch II or III resistance arteries were cleaned of fat and connective tissue, and a segment 1.6 to 2.0 mm in length was removed. In some experiments, the endothelial layer was removed immediately after dissection by friction of a tungsten wire in the vascular lumen. The segment was then mounted on myograph [32] using two tungsten wires inserted through the lumen of the vessel. Mechanical activity was recorded isometrically by a force transducer (Kistler- Morse, DSG BE4). After mounting, the vessel was placed in PSS, kept at 37°C and gassed continuously with 95% O_2_ and 5% CO_2_ (pH 7.4). The vessel was stretched to a length that yields a circumference equivalent to 90% of that given by an internal pressure of 2 mN/mm. Then, the vessel was challenged with 3 µM phenylephrine to elicit contractile responses. The presence of functional endothelium was assessed by the ability of ACh (10 μM) to induce 80% relaxation of vessels pre-contracted with phenylephrine (3 µM). The absence of a relaxation response to ACh was considered evidence that the vessel segments were functionally denuded of endothelium. Phenylephrine concentration (3 µM) was used to induce 70% of its maximum effect in vessel contraction.

### Vasorelaxant activity of triapsin in pre-contracted vessels

The vasorelaxant activity of triapsin was measured in vessels with or without functional endothelium pre-contracted with phenylephrine (3 µM). Triapsin activity was added in increasing cumulative concentrations (8.50 x 10^-15^ M to 0.38 x 10^-9^ M) after stabilization of the phenylephrine response. Experiments were also performed in the presence of L-NAME (300 mM), anti-PAR-2 antibody (SAM-11- Santa Cruz Biotechnology) (10 µg/mL), SBTI (4 µM), alone or pre-incubated with triapsin, or PBS. The antibody PAR-2 (SAM11) is a mouse monoclonal antibody raised against amino acids 37-50 of PAR-2 of human origin, then it can block protease binding and/or the cleavage site of the receptor. L-NAME and anti-PAR-2 antibody were added to the bath 15 min prior to the addition of phenylephrine. SBTI was pre-incubated with triapsin for 15 min on ice. As a control, the effect of SBTI alone was also monitored. Representative tracing of the vasodilation effect of triapsin is shown in result section. 

### Intravital microscopy

Intravital microscopy to assess the effect of triapsin on mouse skin microcirculation was adapted from Soares et al. [33]. Prior to experiments, mice were anesthetized by an intraperitoneal (i.p.) injection of 150 mg/kg ketamine hydrochloride (Cristalia) and 10 mg/kg xylazine (Bayer). To facilitate the microinjection, the mouse ear was fixed on a semi-cylinder glass (a tube divided in half) with a ring of double-sided tape [34], and the mouse temperature was maintained at 37°C with a heating pad (Fine Science Tools Inc., Canada). The injection site on the skin of the ear was examined using an optical microscope (Leica DM500) and was injected using a glass capillary coupled to a micro injector (Nanoject, Drummond). The images (1 photo per minute, 5184 x 2912 pixels) were captured using a digital camera (Canon EOS 600D) and were analyzed using ImageJ software [35]. In each image, a region of the venule near the site of injection was selected and its Raw Integrated Density (sum of pixel values using the tools “Measure Stack” in "Plugins" options) was calculated. The Raw Integrated Density (an estimative of area occupied by blood vessel) values were transferred to GraphPad Prism 6 for graphic construction and analyses.

### Statistical analysis

Data are represented as the mean ± S.E.M. Statistical analysis was performed using Student’s t-test for two-group comparisons. Differences between the concentration-response curves were analyzed statistically by Bonferroni’s test. Significance was set at the p < 0.05.

## Results

### Activity of *Triatoma infestans* saliva on PAR peptides

**Figure 1.**
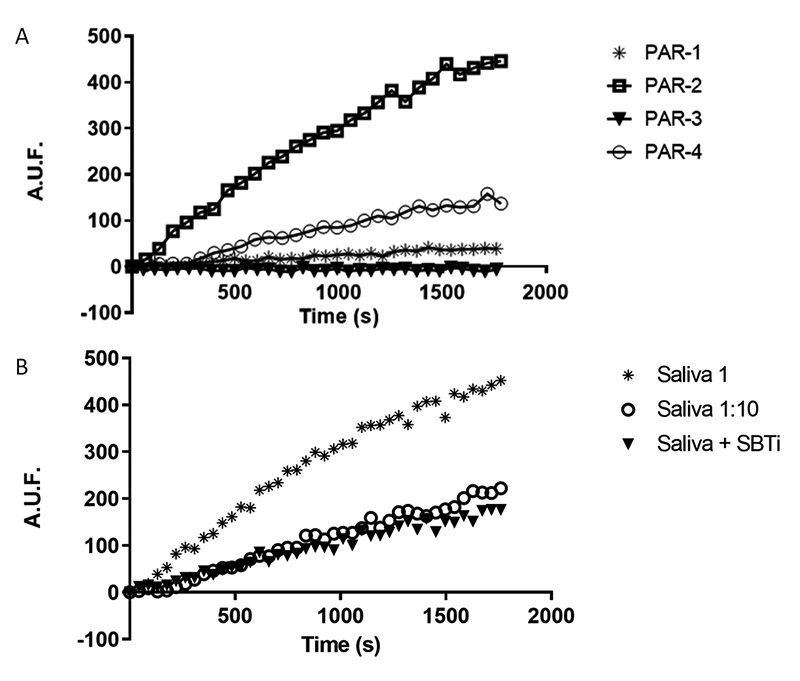
Time course of PAR peptides hydrolysis by *T. infestans* saliva. **(A)** Progress curves of hydrolysis of Abz-PAR-EDDnp (Abz-protease-activated receptor EDDnp) 1, 2, 3 and 4 (50 µM) by 76.8 µg/mL total protein from *T. infestans* saliva. Asterisk: activity of saliva on PAR1 peptide; rectangle: activity of saliva on PAR2 peptide; inverted triangle: activity of saliva on PAR3 peptide; circle: activity of saliva on PAR4 peptide. **(B)** Hydrolysis of PAR-2 peptide (50 µM) at different concentrations of total protein from *T. infestans* saliva (saliva 1 = 76.8 µg/mL; saliva 1:10 = 7.68 µg/mL) and saliva 1 in the presence of SBTI (8 µM). Asterisk: activity of concentrated saliva on PAR2 peptide; open circle: activity of diluted saliva (1:10) on PAR2 peptide; inverted triangle: activity of saliva on PAR2 peptide in the presence of SBTI.

To investigate if triapsin activity has a role in promoting host vasodilation during insect blood feeding by activation of PAR receptors, we initially tested the potential of *T. infestans* saliva to cleave peptides consisting of the sequence spanning the cleavage sites of human PAR receptors. Saliva was incubated with 50 µM FRET peptides at 37°C. Figure 1A represents the time course of PAR peptide hydrolysis and shows a higher preference of the saliva for cleavage of the PAR-2 peptide. PAR-2 peptide hydrolysis was approximately 10-fold and nearly 3-fold higher than PAR-1 and PAR-4 peptide hydrolysis, respectively, and there was no activity toward PAR-3 peptide in the assayed conditions. The PAR-2 cleavage activity was partially inhibited in the presence of the serine protease inhibitor SBTI and it is proportional to the saliva concentration in the assay, as shown in Figure 1B. 

**Figure 2.**
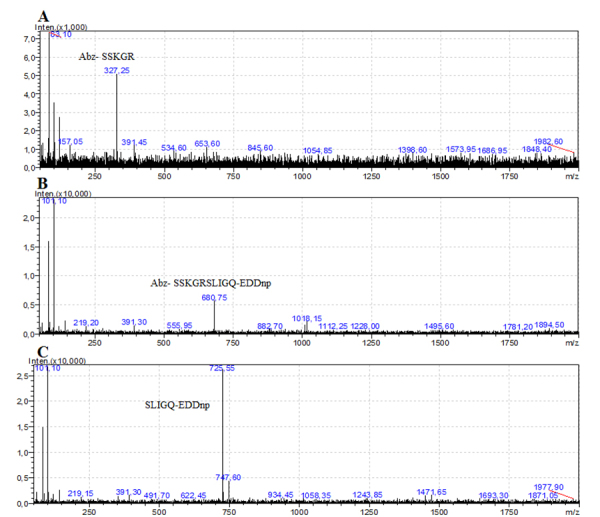
Mass spectrum of PAR-2 cleavage products generated by *T. infestans saliva*. PAR-2 peptide was treated for 30 min at 37 °C with *T. infestans* saliva in 100 mM Tris-HCl buffer (pH 8.0). The amino acid sequences of the fragments are indicated in A, B and C. **(A)** Molecular ion at m/z 327.5 corresponds to m/z of the peptide Abz-SSKGR. **(B)** Molecular ion at m/z 680.75 corresponds to m/z of the peptide Abz-SSKGRSLIGQ-EDDnp. **(C)** Molecular ion at m/z 725.5 corresponds to SLIGQ-EDDnp molecular mass.

The cleavage site in the PAR-2 peptide by saliva was analyzed by mass spectrometry (Figure 2), and it was confirmed to be a single cleavage site that corresponds to the activation site of the PAR-2 receptor (SSKGR↓SLIGQ). In the mass spectrum, the molecular ions at m/z 327,5, m/z 680,75 correspond to m/z of the peptides Abz- SSKGR, Abz- SSKGRSLIGQ-EDDnp, respectively (Figure 2A and B) and m/z 725,5 corresponds to SLIGQ-EDDnp molecular mass (Figure 2C). These results indicate the presence of a specific trypsin-like protease cleaving PAR-2 peptide in the saliva of *T. infestans*. For further investigation, triapsin was partially purified from saliva according to Amino et al. [8].

### Activity of triapsin on PAR peptides

**Figure 3.**
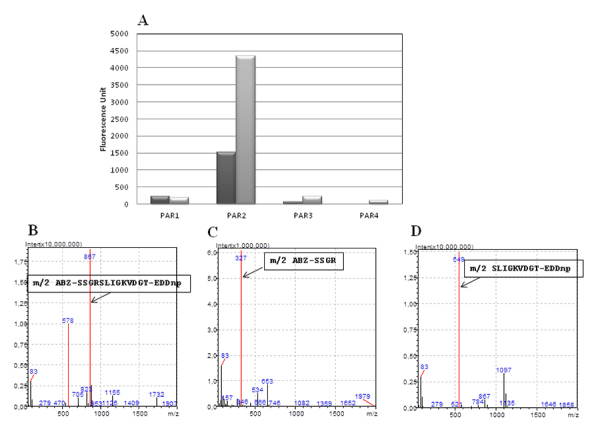
Hydrolysis of Abz-PAR-EDDnp (Abz-protease-activated receptor EDDnp) peptides by purified triapsin. **(A)** Peptide sequences for human PAR-1, -2, -3, and -4 activation: PAR-1_TLDPR↓SFLLRN, PAR-2_SSKGR↓SLIGKVDGT, PAR-3_TLPIK↓TFRGAPPNS, PAR-4_ LPAPR↓GYPGQVCAN were incubated with triapsin purified on Superdex G 75. Assays were performed with triapsin purified on Superdex G75 and 125 μM each peptide in 100 mM Tris-HCl buffer pH 8.0 at 37°C in a Microplate Spectrophotometer (Gene5_BioTek® Instruments) and read at 320 nm and 420 nm for excitation and emission, respectively. **(B)** Molecular ion at m/z 867 corresponds to m/2 of the whole peptide (Abz-SSKGRSLIGKVDGT-EDDnp). **(C)** Molecular ion at m/z 327.0 corresponds to m/2 of the fragment Abz-SSKGR from PAR-2 incubated with triapsin. **(D)** Molecular ion at m/z 549 corresponds to m/2 of the fragment SLIGKVDGT-EDDnp from PAR-2 incubated with triapsin.

Samples of purified triapsin activity (Additional file 1) were incubated with 125 µM FRET peptides at 37°C. Figure 3A represents PAR peptide hydrolysis when incubated with triapsin activity for 30 min and 22 h. The hydrolysis profile was similar to the one from saliva, showing that triapsin activity presented high preference for cleavage of the PAR-2 peptide. 

The cleavage site of triapsin activity in the PAR-2 peptide was also confirmed by mass spectrometry (Figure 3B to 3D and Additional file 2) and it was confirmed to be a single cleavage site that also corresponds to the activation site of the PAR-2 receptor. In the mass spectrum, the molecular ions at m/z 867, m/z 327 and m/z 549 correspond to m/2 of the peptides Abz- SSKGRSLIGKVDGT -EDDnp (Figure 3B), Abz- SSKGR (Figure 3C), and SLIGKVDGT -EDDnp molecular mass (Figure 3D). These results indicate the presence of a specific trypsin-like protease cleaving PAR-2 peptide in the saliva of *T. infestans*. 

**Figure 4.**
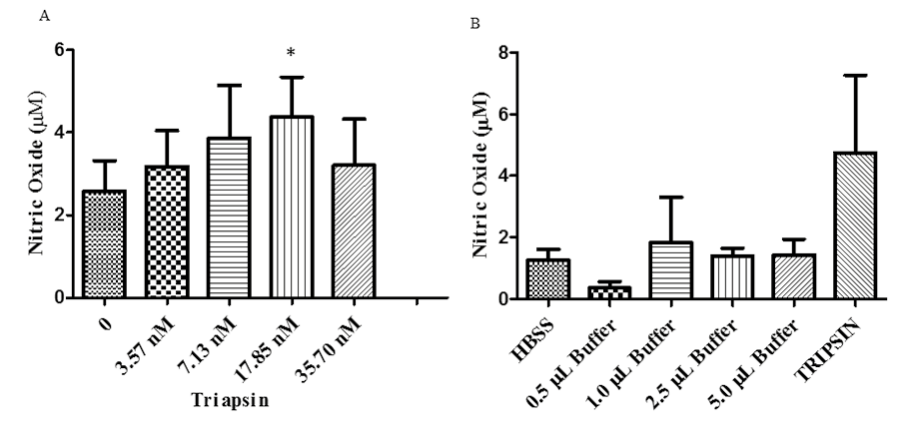
Analysis of triapsin-induced endothelial NO release by cultured HUVECs (human umbilical vein endothelial cells). **(A)** Dose-dependent NO release by cultured HUVECs induced by triapsin quantified by the fluorescent dye for nitrite/nitrate (DAN). Data show the mean ± S.E.M. from five experiments with 5 x 10^3^ cells. One-way ANOVA with Dunnett's multiple comparisons test, followed by a ratio-paired t-test were used to calculate differences to control; *p < 0.05. **(B)** Stimulation of NO release by HUVECs measured in the presence of different amount of triapsin-buffer in the same volumes used for the triapsin dose-dependent curve (n = 3). One-way ANOVA with Dunnett's multiple comparisons test was used to calculate differences. Trypsin (50 nM) was used as a positive control.

### Triapsin-induced NO release by human umbilical vein endothelial cells (HUVECs)

Animal studies have shown that NO is a primary mediator of vascular tone in many species [36-38]. In this work, the release of NO by cultured endothelial cells was examined upon triapsin stimulation using a fluorometric assay for the quantification of nitrite/nitrate [30]. Cell viability was assessed using a MTT assay (n = 3) and showed that treatment with triapsin did not reduce significantly (p > 0.05) cell viability compared to the untreated control (HBSS only) (data not shown). 

Figure 4A shows the concentration dependence of triapsin activity-induced NO synthesis by HUVECs (5 x 10^3^ cells). A significant (p < 0.05) increase in NO production was observed compared to the control at 17.85 nM of the protease activity, corresponding to a maximum effect of 5.6 ± 3.1 µM of released NO. Control experiments (n = 3) were performed using the same volumes of enzyme buffer and showed no statistical difference (p > 0.05) related to the untreated control (HBSS only) (Figure 4B). Trypsin (50 nM), which is expected to induce NO release by similar mechanism as triapsin activity by cleaving PAR-2 peptides [10] was used as a positive control and induced the release of 5.49 ± 0.81 μM (mean ± SEM) (Figure 4). Acetylcholine (10^-5^ M) was used to determine the maximum effect of NO production and induced release of 16.68 ± 11.90 μM (mean ± SEM). 

### Triapsin activity induces endothelium-dependent vascular relaxation by activating the PAR-2 receptor

**Figure 5.**
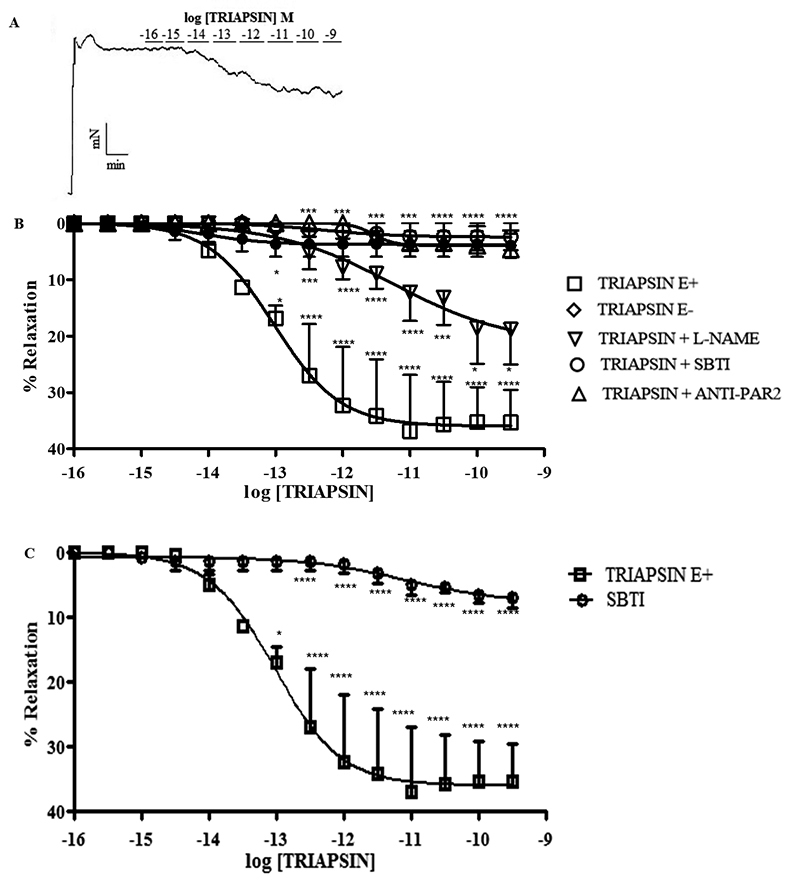
Triapsin induces relaxation of mouse mesenteric artery that is mediated by endothelial PAR-2. **(A)** Representative tracing depicting the concentration-dependent vasodilation effect of triapsin in endothelium-containing mesenteric arteries rings from Swiss mice pre-contracted with phenylephrine (3 µM). **(B)** Shows the mean ± S.E.M. of the vasodilation effect of triapsin in endothelium-containing vessels in the absence (triapsin E+) or in the presence of NOS inhibition (triapsin + L-NAME) or PAR-2 inhibition (triapsin + anti-PAR-2). The concentrations of L-NAME and anti-PAR used were 300 µM and 10 µg/mL, respectively. A control with the serine protease inhibitor SBTI (4 µM) was included. The requirement for an intact endothelium for triapsin-induced relaxation was tested in endothelium-denuded vessels (triapsin E^-^). Two-way ANOVA with Bonferroni’s multiple comparisons test was used to analyze the differences. **p < 0.01; ***p < 0.001, ****p < 0.0001. diamond: triapsin E-; rectangle: triapsin E+; inverted triangle: triapsin + L-NAME; empty circle: triapsin + SBTI; triangle: triapsin + ANTI_PAR2. (**C)** Curves show mean ± S.E.M. of the vasodilation effect of triapsin in endothelium-containing vessels in the absence (rectangle) of SBTI and the effect of SBTI (circle). SBTI concentration was 4 µM. Two-way ANOVA with Bonferroni’s multiple comparisons test was used to analyze the differences. *p < 0.05; ****p < 0.0001.

We examined if triapsin activity is capable of inducing vasodilation via PAR-2 activation. Triapsin (10^-16^ to 10^-9^ M) was applied cumulatively to mouse mesenteric artery rings pre-contracted with phenylephrine (3 µM). Figure 5A shows a typical recording of vasodilation caused by triapsin activity. As seen in Figures 5A and 5B, the maximum relaxation response, approximately 35%, was induced by triapsin (10^-11^ M). This response was not observed in endothelium-denuded preparations, and it was partially but significantly (p < 0.001) attenuated when the vessel was pre-treated with L-NAME (300 µM) and stimulated with increasing concentrations of triapsin activity from 0.385 x 10^-12^ M to 0.385 x 10^-9^ M (Figure 5B). Furthermore, the relaxation response induced by triapsin activity was completely inhibited in the presence of an anti-PAR-2 antibody (10 µg/mL) (Figure 5B), showing that the triapsin activity relaxation effect involves different pathways of vasomotor modulation triggered by endothelial PAR-2, as shown by Kawabata et al. [23] in rat mesenteric artery. Controls for this experiment included the enzyme vehicle (PBS) that induced no effect (n = 5) and SBTI (4 µM), a serine-protease inhibitor that abrogated the triapsin activity-induced relaxation response at effective concentrations (n = 2). SBTI alone did not induce any response (n = 3) (Figure 5C). 

### Intravital microscopy analysis of vessel diameter alterations induced by triapsin activity

**Figure 6.**
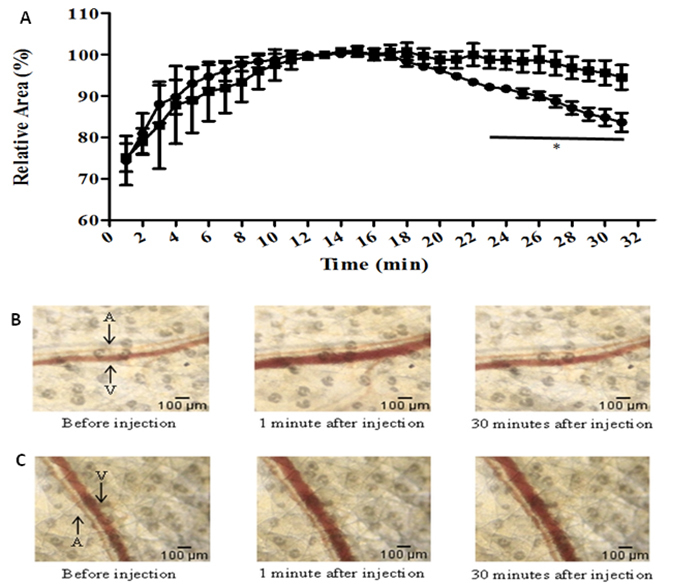
Effect of triapsin in mouse skin microcirculation. **(A)** Mouse ear venular modulation was analyzed upon triapsin stimulation by intravital microscopy. The control consists of the same volume of PBS pH 7.4. The graphic represents the mean increase in venular area upon injection of triapsin (rectangle) (n = 4) or PBS (circles) (n = 5). The maximum vasodilation obtained was considered 100%. Two-way ANOVA with a post t-test (unpaired t-test) were used to analyze the differences (*p < 0.05). The mean ± SEM is shown. **(B)** Diameter variation of mouse ear venules at indicated time points after intradermal microinjection of PBS (pH 7.4) (69 nL). **(C)** Diameter variation of mouse ear venules at indicated time points after intradermal microinjection of triapsin (69 nL; 15.1 (M). In the images, arteriole (A) and venule (V) are indicated by arrows.

Image analysis of mouse skin microcirculation upon injection of triapsin activity allowed the graphical representation of vessel dilation shown in Figure 6A. The microinjection of 69 nL of triapsin (15.1 (M) or PBS in ear skin elicited a reflex vasodilation, showing the maximum value after ~13 minutes. Triapsin activity was able to maintain the vasodilation practically unchanged for up to 30 min of observation, while, in the same period in the assays with PBS, a gradual reduction of vessel area was seen until it reached values similar to the ones before the microinjection (Figure 6B and 6C). 

## Discussion

*T. infestans*, like other hematophagous arthropods, possesses a variety of molecules to counteract host hemostatic responses [3-5, 39-41]. Regarding the ability to induce vasodilation, some hematophagous insects take advantage of the vasodilator properties of NO, including the triatomine *Rhodnius prolixus* and the cimicid *Cimex lectularius* [42,43]. Those insects express and secrete in their saliva a group of heme proteins (nitrophorins) that store and deliver NO in host blood vessel during feeding. *T. infestans* has no nitrophorins. Ribeiro et al. [43] showed that extracts of the salivary gland of *T. infestans* induce vasodilation in an endothelium-dependent manner. However, thus far, this activity had not been identified or characterized from the saliva of this insect; in addition, a mechanism has not been proposed for *T. infestans* induction of vasodilation during blood feeding. 

In this work either *T. infestans* saliva or the fraction of *T. infestans* saliva containing triapsin activity showed high preference toward PAR-2 over PAR-3 peptide. Saliva also showed some activity toward PAR-1 and PAR-4 peptides. These results are in accordance with the specificity of triapsin amidolytic activity described by Amino et al. [8] that demonstrated a high specificity of purified triapsin for arginine and no activity for lysine at the P1 site. The single scissile bond in PAR2 peptide, confirmed by mass spectrometry, corresponds to the activation site of PAR2 peptides within the N-terminal exodomain by trypsin and mast cell tryptase [10, 44,45]. Despite the presence of potential sites for cysteine- and serine-proteases in this peptide [46], the single cleaved site weakens the hypothesis of serine proteases other than triapsin in this preparation of *T. infestans* saliva. In agreement, a unique proteolytic activity that migrated at 40 kDa was shown from *T. infestans* saliva by Amino et al. [8]. 

It has been suggested that trypsin-like proteases from saliva of hematophagous hemiptera might play specific roles, processing specific substrates, differently to typical digestive trypsin-like proteases, counteracting hemostatic processes of the host as an evolutionary adaptation that allows the success of the blood feeding behavior [47,48]. Our results are in accordance, and strongly indicate that triapsin activity is responsible for the PAR peptide hydrolysis activity presented in *T. infestans* saliva, which led us to investigate the potential vasomotor modulation activity of triapsin activity via activation of the PAR-2 receptor in the membrane of endothelial cells. 

Endothelial cells can mediate vasodilation via three different mechanisms: NO, endothelium-derived hyperpolarizing factor (EDHF) and prostaglandins ([49]; for review). Activation of the PAR-2 receptor has been associated with all these components [24-26, 50,51]. 

We demonstrated that triapsin activity could trigger NO release by cultured endothelial cells in a significant and dose-dependent manner. The activation of endothelial NO synthase with consequent NO release is one of the pathways triggered by PAR-2 in the membrane of endothelial cells to promote endothelium-dependent vasorelaxation [52-54]. As *T. infestans* is a vessel feeding insect [55], results indicate that salivary triapsin activity vasodilation is mediated by the PAR-2 cleavage which induces NO production and release by endothelial cells of vessels at the biting site. This is a mechanism different from hematophagous hemipterans that express nitrophorins [42,43] that has not yet been described for hematophagous insects. 

Triapsin activity showed a potent vasodilator effect on mouse mesenteric arteries (EC_30_ = 10^-12^ M), as well as on mouse skin microcirculation and the endothelial-dependence of this effect was confirmed by the absence of relaxation in denuded endothelium. 

Endothelial NO release seems to contribute in the endothelial-dependent relaxation activity of triapsin activity, which was verified by the residual effect of triapsin on mouse mesenteric artery in the presence of the NO synthase inhibitor L-NAME. This result is in accordance with the McGuire et al. [26,43] study on this type of vessel. The authors demonstrated that multiple mechanisms are involved in vascular smooth muscle relaxation by the activation of PAR-2 in this vessel that included partial contributions of NO and cyclic GMP as well as EDHF-mediated mechanisms. 

Triapsin activity seems to be highly selective for PAR-2 receptor activation. PAR-2 dependence of triapsin activity for induction of relaxation was confirmed by abrogation of this relaxation in the presence of the anti-PAR-2 antibody. In contrast, bovine trypsin was able to induce some relaxation of mesenteric arterioles of PAR-2 (-/-) mice at concentrations not selective for PAR-2 over PAR-1 activation [26].

PAR-2 expression and activation in vein and venules have been reported [56-58]**,** and they are associated with the relaxation of human venous beds with contributions of both NO and prostanoid mediators [59]. We did not examine whether the effect of triapsin activity on the mouse ear venular diameter (Figure 6) was mediated by PAR-2 receptors, but the significant increase in the venular area induced by the protease compared to the negative control indicates that it also plays a role in this vessel. 

Although the mechanism of dilation of the endothelium can vary between species or within the same species [23-26,60], PAR-2 seems to be present in the endothelium of most arteries and induces relaxation upon activation [23]. Then, by activating PAR-2 receptor, triapsin activity can potentially induce host vasodilation during insect blood feeding.

## Conclusion

In this article, we showed that triapsin activity induces vasodilation via PAR-2 activation and we described for the first time a protease activity from the saliva of a hematophagous animal acting as a vasodilator.

### Abbreviations

Abz: o-aminobenzoic acid; EDDnp: ethylenediamine-2,4-dinitrophenyl; L-NAME: L-N^G^-nitroarginine methyl ester; PARs: protease activated receptors; SBTI: soybean trypsin inhibitor.

## Supplementary material

The following online material is available for this article:

Additional file 1. Triapsin purification.

Additional file 2. Mass spectrum of PAR-2 cleavage products generated by triapsin purified by hydrophobic interaction on Source 15 PHE (Phenyl) column
