# Supplementary material for: Proteolytic activity of Triatoma infestans saliva associated with PAR-2 activation and vasodilation
Source: J Venom Anim Toxins Incl Trop Dis. 2021 Mar 8;27:e20200098. doi: 10.1590/1678-9199-JVATITD-2020-0098 (PMC7939238; doi:10.1590/1678-9199-JVATITD-2020-0098)
Supplement: Additional file 1. [file 1678-9199-jvatitd-27-e20200098-s1.pdf]

*J Venom Anim Toxins incl Trop Dis*
https://doi.org/10.1590/1678-9199-JVATITD-2020-0098

# "Supplementary Material to "Proteolytic activity of *Triatoma infestans* saliva associated with PAR-2 activation and vasodilation"

**Additional file 1.** Triapsin purification.

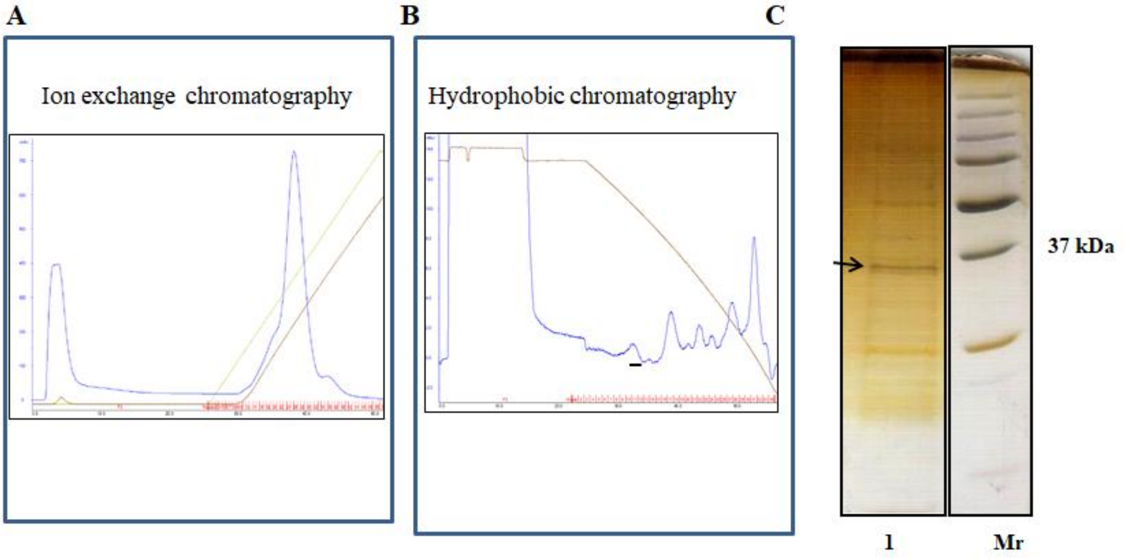


**(A)** Ion exchange chromatography. An amount of 50 µL of *Triatoma infestans* saliva diluted in water was applied to a HiTrapQ column. Proteins were eluted by NaCl gradient (0-1 M). (**B)** Hydrophobic chromatography. Active material from HiTrap Q, 148 µU, was pooled and adjusted to 1.7 M ammonium sulfate, and applied to a Source PHE column. Proteins were eluted with buffer B (tris 0.05M buffer, pH 8.0) and active fractions were pooled and concentrated totaling 46.5 µU of triapsin. In this panel a bar is indicating the active fractions. **(C)** Silver-stained SDS-PAGE (12%). Purified triapsin from saliva of *T. infestans*: 1.3% of the pool from hydrophobic chromatography on Source PHE column. Mr: protein molecular markers.
