# Supplementary material for: Proteolytic activity of Triatoma infestans saliva associated with PAR-2 activation and vasodilation
Source: J Venom Anim Toxins Incl Trop Dis. 2021 Mar 8;27:e20200098. doi: 10.1590/1678-9199-JVATITD-2020-0098 (PMC7939238; doi:10.1590/1678-9199-JVATITD-2020-0098)
Supplement: Additional file 2. [file 1678-9199-jvatitd-27-e20200098-s2.pdf]

J Venom Anim Toxins incl Trop Dis
https://doi.org/10.1590/1678-9199-JVATITD-2020-0098

"Supplementary Material to "Proteolytic activity of *Triatoma infestans* saliva associated with PAR-2 activation and vasodilation"

Additional file 2. Mass spectrum of PAR-2 cleavage products generated by triapsin purified by hydrophobic interaction on Source 15 PHE (Phenyl) column.

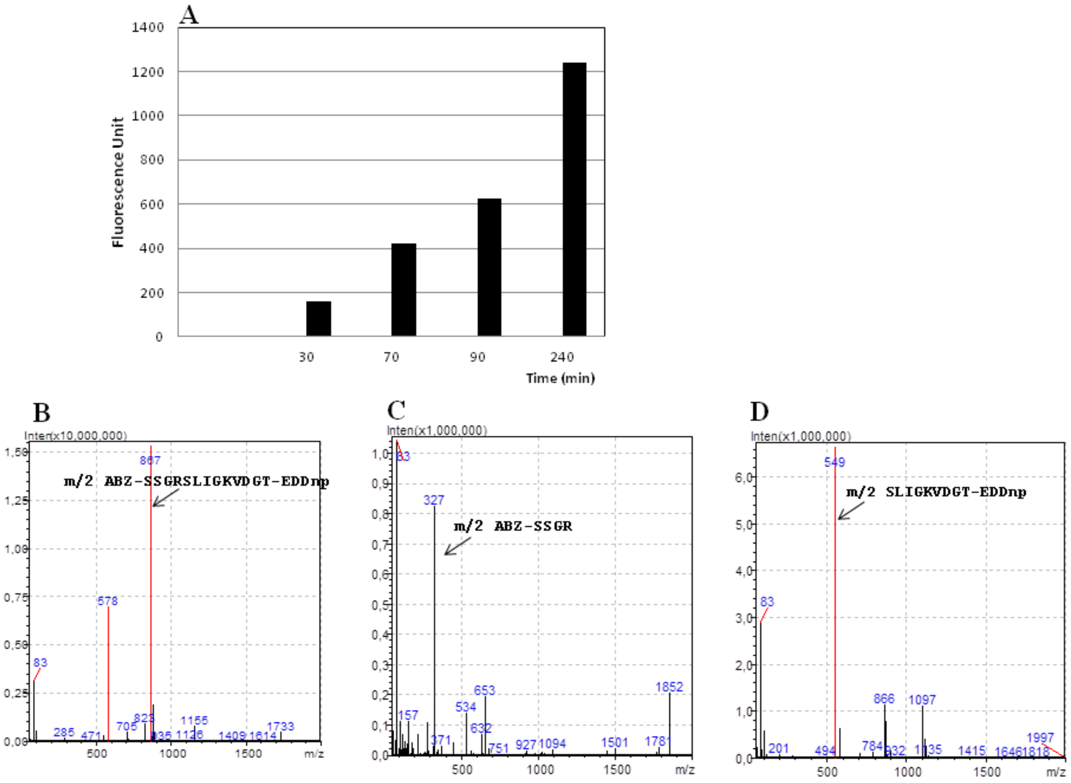

(A) PAR-2 peptide was treated 4h at 37°C with triapsin in 100 mM Tris buffer pH 8.0 and the products of hydrolysis was read at 320 nm and 420 nm for excitation and emission, respectively, in a Microplate Spectrophotometer (Gene5_BioTek® Instruments), as well as the whole peptide, were submitted to (MALDI-TOF) mass spectrometry. **(B)** Molecular ion at m/z 867 corresponds to m/2 of the whole peptide (Abz-SSKGRSLIGKVDGT-EDDnp). **(C)** Molecular ion at m/z 327.0 corresponds to m/2 of the fragment Abz-SSKGR. **(D)** Molecular ion at m/z 549 corresponds to m/2 of the fragment SLIGKVDGT-EDDnp.
